# Adjuvant Radiotherapy and Breast Cancer in Patients with Li-Fraumeni Syndrome: A Critical Review

**DOI:** 10.3390/cancers17071206

**Published:** 2025-04-01

**Authors:** Adnan Shrebati, Pierre Loap, Youlia Kirova

**Affiliations:** Department of Radiation Oncology, Institut Curie, 75005 Paris, France; adnan.shrebati@curie.fr (A.S.); pierre.loap@curie.fr (P.L.)

**Keywords:** Li-Fraumeni syndrome, breast cancer, radio-induced malignancies, adjuvant radiotherapy, tp53

## Abstract

**Background/Objectives**: Li-Fraumeni syndrome (LFS) is a disease caused by a germline mutation of the tp53 gene that predisposes the individual to various malignancies, including breast cancer. Its impact on treatment modalities in oncology remains poorly studied. In this critical review, we aimed to retrieve and analyze available data concerning adjuvant radiotherapy in early breast cancer for LFS patients, as well as the current guidelines. **Methods**: We reviewed articles with LFS patient cohorts that reported secondary malignancy rates after breast adjuvant radiotherapy. Rates of recurrence, when available, were also included. Furthermore, we discussed contemporary radiobiological evidence and guidelines on the subject. **Results**: Six retrospective studies were reviewed. Five reported much higher rates of secondary malignancies compared to the general population. Additionally, there was no clear trend toward increased locoregional control after adjuvant radiotherapy. Radiobiological data suggest increased radioresistance and radiosensitivity within p53-mutated cells. Guidelines recommend avoiding radiotherapy in LFS patients when possible. **Conclusions**: Currently, there is no standard treatment or cure for LFS or a germline variant of the TP53 gene. With few exceptions, cancers in people with LFS are treated the same way as cancers in other patients, but research continues into the best way to manage cancers involved in LFS.

## Simple Summary

Patients suffering from Li-Fraumeni syndrome (LFS) are often thought to be predisposed to radiation-induced malignancies. Radiobiological data seems to indicate an association between tp53 mutations and both radio resistance and radio susceptibility. Most retrospective cohorts concerning adjuvant breast cancer radiation therapy indicate an increased prevalence of radio - induced malignancies in Li-Fraumeni syndrome patients. Current guidelines support alternative treatment modalities whenever available and alternative diagnostic methods without radiation for the screening of different type of malignancies in this population of patients.

## 1. Introduction

Li-Fraumeni syndrome is an autosomal dominant disorder affecting one in every 500 to 20,000 individuals [1], caused by a germline mutation of tp53 coding for p53, which is often called the guardian of the genome due to its role in regulating gene damage and inducing cell cycle arrest, up to apoptosis if the damage crosses a certain threshold [2]. Described by Li and Fraumeni in 1969, it is characterized by a high risk of developing a wide range of tumors during one’s lifetime, including but not limited to soft tissue sarcoma, choroidal plexus carcinoma, high-grade glioma and breast cancer. The all-cancer type lifetime risk ranges from 73% to 100% [3], including an 85% risk of developing breast cancer before 65 years of age among female patients. Concerning the latter, it is estimated that 5 to 8% of patients with a diagnosis of breast cancer before the age of 30 are a consequence of a germline tp53 mutation [4].

One of the main axes of treatment for breast cancer is adjuvant radiotherapy after conservative surgery or total mastectomy, greatly reducing the risk of local recurrence and increasing overall survival [5]. However, several cases have been reported in the literature linking radiotherapy with a higher risk of radio-induced malignancies (RIM), asking the question of whether radiotherapy remains a net benefit for these patients [6]. Nevertheless, the data remain limited. The aim of this review is to provide some elements of response using the information available in the literature concerning the interactions of p53 with radiation damage as well as the different patient cohorts that have been published up to this day.

## 2. Materials and Methods

The research was realized using a search in all published papers in PubMed with keywords: Li-Fraumeni syndrome; breast cancer; radio-induced malignancies; adjuvant radiotherapy; tp53.

### 2.1. P53 and Its Interactions with Radio Sensitivity

Tp53 is a tumor suppressor gene that aids in preserving the integrity of the genome through several mechanisms. Coding for the p53 protein, this transcription factor activates following the emergence of gene damage-induced stress factors, therefore promoting the transcription of multiple proteins notably involved in cell cycle arrest, gene repair and apoptosis. This is achieved by upregulating the expression of p21 (also known as Cip1), a cyclin-dependent kinase inhibitor (CKI) [2]. p21 inhibits the activity of cyclin-dependent kinases (CDKs), particularly CDK2 and CDK4/6, which are required for the G1/S transition. Several DNA repair pathways are then triggered, such as base excision repair (BER) and nucleotide excision repair (NER). P53 may also induce arrest in the G2/M phase, allowing for homologous recombination (HR) to repair double strand breaks with high fidelity [7].

The existing literature provides conflicting evidence on whether tp53 mutations are predictive of increased resistance or sensitivity to adjuvant/neoadjuvant radiotherapy. Rozan et al. [8] found no predictive value regarding tumoral p53 expression and radio sensitivity among breast cancer patients, and Silvestrini et al. [9] found no difference in local tumor recurrence after conservative surgery followed by radiation therapy between early breast cancer patients with tumors expressing vs. not expressing p53. However, McIlWrath et al. [10] reported a negative correlation between murine cell radio sensitivity and tp53 mutations (tp53m), as G1/S cell cycle arrest was positively associated with radiation response [11]. Bergh et al. found a clear negative link between tumoral tp53 mutations and breast cancer patient responses to adjuvant radiotherapy [12].

Several papers have researched the possible underlying processes explaining such observations. Boyle et al. [13] found a decreased radiation-induced G1/S cell cycle among fibroblasts cultured from Li-Fraumeni patients, suggesting a higher threshold of DNA double strand breaks (DSB) needed to cause blockage, which would lead to lower radio sensitivity but possibly a higher radio susceptibility, meaning an increased risk of cells surviving with DNA damage allowing the emergence of secondary radiation-induced malignancies in the long term.

Anbalagan et al. [14] studied the relationship between fraction size sensitivity, p53 expression and non-homologous end joining repair (NHEJ). The repair of DSBs by NHEJ plays a crucial role in determining the radio sensitivity of cells, especially those with low proliferative indices, such as muscle, heart, and brain cells. These cells, which are primarily in the G0 phase of the cell cycle, exhibit significant sensitivity to radiation doses per fraction due to their reliance upon NHEJ, its fidelity decreasing with the amount of DNA lesions present. This characteristic is also observed in certain cancer types, including breast and prostate cancers.

The authors observed that cells with wild-type p53 (p53wt) are sensitive to dose per fraction, whereas p53-mutant (p53m) cells show resistance to dose per fraction and fail to recover between fractions. This lack of sensitivity to hypo-fractionation could be a result of their higher proliferative index and increased arrest at the S/G2 stage leading to an intensified use of HR for DSBs, which is not sensitive to dose per fraction due to its high fidelity. These results suggest that modern hypo-fractionated radiotherapy schemes could be less effective when treating patients with tp53 mutant tumors or with germline tp53 mutations.

### 2.2. Radio-Induced Tumors and Increased Risk Among Li-Fraumeni (LFS) Patients

#### 2.2.1. General Population

About 50% of cancer patients will receive radiation therapy at some point during their care, in either a curative or a palliative context. After surviving their primary malignancy, 17 to 19% of patients will develop a secondary malignancy, this risk being attributed to genetic predisposition, lifestyle choices, and treatment modalities [15]. Radiotherapy only accounts for 5% of total treatment-related second malignancies or about 8 to 10% of post-radiotherapy second cancers [16]. For breast cancer patients, this amounts to a relative risk of 1.22 of developing a second non-breast cancer, particularly sarcomas, lung and esophageal cancer [15]. The main histology of these radiation-induced malignancies (RIM) appearing within the field of irradiation are sarcomas, with carcinomas and leukemia also being at risk within tissues receiving small amounts of irradiation [17,18]. Soft tissue RIMs have a latency period of about 10 to 60 years, while radiation-induced leukemia generally appears 5 to 10 years after exposure [19].

For breast cancer patients, Kirova et al. [20] reported the experience of the Institut Curie of 13,472 breast cancer patients treated with radiation therapy between 1981 and 1997 to evaluate the risk of radiation-induced sarcomas (RIS) using the Cahan criteria [21], as follows:

1. History of radiotherapy;
2. Asymptomatic latency period of several years;
3. Occurrence of sarcoma within irradiated field;
4. Histological confirmation of the sarcomatous nature of the lesion.

In the Institut Curie we found a total of 35 cases, 27 meeting the Cahan criteria. The cumulative incidence was 0.27% at 10 years and 0.48% at 15 years, confirming a relatively low risk of radiation-induced sarcomas in the general population (RIS). However, Grantzau et al. [22] communicated in their meta-analysis an RR of 2.53 for developing second sarcoma after breast adjuvant radiotherapy, which could be an important element to consider within populations with an already high frequency of sarcomas.

#### 2.2.2. Li-Fraumeni Patients

Murine models have long supported the notion of increased risk of RIMs among Li-Fraumeni patients due to a higher threshold of activation for G1/S cell cycle arrest, leading to an increased survival rate among irradiated cell lines and thus contributing to carcinogenesis.

Mitchel et al. [23] published an upper dose threshold below 100 mGy for radiation-induced tumors in cancer-prone radiation-sensitive tp53 heterozygous mice. Kasper et al. [24] found in murine models for LFS an increased risk of secondary malignancies after exposure to X-rays and genotoxic chemotherapies such as etoposide compared to tp53 wild-type mice (Hazard Ratio of 4.4), with reduced life expectancy.

Clinical data on LFS patients also seem to support this higher risk of RIMs. Heyn et al. [25] found a higher cumulative incidence of RIMs in children that underwent radiotherapy for rhabdomyosarcoma, particularly when there was a family history suggestive of neurofibromatosis or Li-Fraumeni syndrome. The risk was further elevated if alkylating agents were administered concurrently.

In one of the larger available cohorts, Bougeard et al. [26] reviewed the data concerning 1730 patients with a history suggestive of LFS, of which 64 received radiotherapy at some point in their care. Among these, 19 (30%) developed one or more secondary malignancies within the irradiated field with a mean timing of 10.7 years after irradiation. Similarly, Suri et al. [27] found a 48% risk of secondary malignancy within a previously irradiated field in a cohort of 23 LFS patients. However, even here the clinical data are conflicting, as in a cohort of 40 LFS patients of whom 14 patients received radiotherapy, Hendrickson et al. [28] did not find an increased risk of secondary malignancy. Still, the median 4.5-year duration of follow-up was perhaps too short to notice a difference.

There is a problem of heterogeneity, such as differences in patient populations, radiotherapy protocols, follow-up duration, and LFS diagnostic criteria.

## 3. Results

### 3.1. Retrospective Studies Concerning Breast Cancer Among Li-Fraumeni Patients

Concerning specifically LFS breast cancer patients treated with adjuvant radiotherapy, we identified six cohorts [29,30,31,32,33,34], presented in Table 1.

All studies found an over-representation of HER2-amplified tumors, consistent with past observations [35]. All cohorts were of limited size with the exception of Sandoval et al., with 227 patients total [34]. Median follow-up ranged from 4.375 years [30] to 12.5 years [31], and longer follow-up durations did not find a higher proportion of RIMs compared to the cohorts with shorter durations.

Not all studies presented differences in loco regional recurrences between irradiated and non-irradiated patients, but those that did seemed to tend towards a reduction in risk.

RIM rates varied widely from cohort to cohort, but they all found an increased rate compared to the general population with the exception of Alyami et al., who did not find any. For the others, the proportion varied from 5.6% [31] to 30% [29].

Median age at diagnosis ranged from 30 years [29] to 39 years [30]. Petry et al. reported that most of their patients (87%) were unaware of their LFS diagnosis at the time of their breast cancer treatment.

Unfortunately, most of the studies are case reports without prospective records or case-control analysis. Because of the heterogeneity of the populations and, in some cases, the small number of patients, we present the results of published studies in Table 1.

### 3.2. Current Guidelines

Current guidelines recommend the use of the revised 2015 Chompret criteria to orient the screening of patients suspected of LFS [26]:

1. Proband with tumor belonging to the LFS spectrum before age 46, and at least one first degree or second-degree relative with LFS tumor (except breast cancer if proband was breast cancer) before age 56 with multiple tumors;
2. Proband with multiple tumors (except multiple breast tumors), two of which belong to the LFS tumor spectrum and the first of which occurred before age 46;
3. Breast cancer before age 31;
4. Patients with adrenocortical carcinoma, choroid plexus tumor, or rhabdomyosarcoma of embryonal anaplastic subtype, irrespective of family history.

As for treatment guidelines regarding radiotherapy, Bergom et al. [36] considered LFS to be one of the conditions predisposing towards a higher rate of RIMs, while leaving it to practitioners’ discretion to discuss interdisciplinarily whether to avoid or treat with radiation therapy.

ASCO 2020 guidelines [37] recommend with moderate strength avoiding adjuvant breast radiotherapy whenever possible, opting for radical mastectomy instead of conservative surgery; postmastectomy RT should only be considered in patients with significant risk of locoregional recurrence.

Finally, Frebourg et al. [38] underlines the importance of genetic screening before treatment initiation if there are clinical elements causing suspicion of LFS. The proposed surveillance regimen in adults would be an annual clinical examination, whole body MRI, breast MRI in females from 20 to 65 years and brain MRI until 50 years. For treatment, priority should be given to surgical or ablative treatments, avoiding radiotherapy when possible and using preferably non-genotoxic chemotherapies.

## 4. Discussion

In this paper, we reviewed the different cohorts available in the literature studying adjuvant breast cancer radiotherapy and its potential risk of RIMs in patients suffering from LFS. Most of them suggest a much higher risk of secondary malignancy compared to other patients, with the exception of Alyami et al. [32], and without a clear trend towards increased locoregional control emerging after irradiation. Still, the data remain of relatively poor quality due to their retrospective nature, the lack of control groups and the limited number of patients available, but this is to be expected considering the rarity of LFS. Median follow-up durations were perhaps too short to effectively measure higher rates of secondary malignancies, as the latency period for RIMs in the general population extends beyond 10 years post exposure [19].

At the same time, in case of localized tumors, the patient should be informed that the alternative to radiotherapy is total mastectomy and the treatment decision must be shared among the radiation oncologist, surgeon and the patient. On the other hand, in cases of aggressive and advanced disease, radiation therapy should be discussed and proposed to patients because its benefits are already established in large prospective phase III studies. This represents the official recommendation. When radiation cannot be avoided, hadron therapy is posited as a valuable alternative.

Another concerning data point was the high proportion of patients treated before their LFS diagnosis could be established [33]. This is worrying, especially when guidelines advise against radiotherapy and genotoxic treatment modalities. A wider implementation of the Chompret criteria in clinical practice could help to limit this issue.

The lack of clear evidence of increased locoregional control post adjuvant radiotherapy raises other questions regarding treatment guidelines. LFS breast cancer patients suffer from elevated risks of ipsilateral and contralateral recurrence, with rates approaching 20% at 7 years for both [39]. Consequently, bilateral radical mastectomy could be a therapeutic option to evaluate in a patient-per-patient situation.

Moreover, other genotoxic treatment modalities and their respective roles in secondary malignancies should be reviewed. Kirova et al. [40] reported that chemotherapy rather than radiotherapy was considerably more associated with secondary leukemia, and vice versa for sarcomas. This was not the case for thyroid and head and neck cancer. Murine models [24] also support a more careful choice of chemotherapy molecules when treating LFS patients. This should also inform researchers when attributing subtypes of secondary malignancy to past treatment modalities. Additionally, genetic testing of secondary tumors could aid in this identification endeavor [41].

When radiation cannot be avoided, hadron therapy is posited as a valuable alternative. Indeed, thanks to its favorable dosimetric characteristics, the reduction in irradiated volume could offer reduced risk of RIMs in LFS patients, as has been previously elaborated in the literature [42,43]. Radiobiological data also seem to indicate greater sensitivity to hadron therapy in tp53m tissues due to its higher linear energy transfer [16]. The current consensus is:

**Radiation therapy (RT):** RT of the intact breast is contraindicated.

Postmastectomy RT should only be considered in patients with a significant risk of locoregional regional recurrence.

**Surgical therapy:** A mastectomy is the recommended therapeutic option.

**Systemic drug therapy:** Avoid cytotoxic anticancer drugs that induce DNA damage if possible. PARP inhibitors: there is insufficient evidence for moderately penetrant genes, including TP53.

**Diagnostic imaging**: Avoid radiation exposure (e.g., ultrasound, MRI). MRI, magnetic resonance imaging; RT, radiation therapy.

## 5. Conclusions

Currently, there is no standard treatment or cure for LFS or a germline variant of the TP53 gene. With few exceptions, cancers in people with LFS are treated the same way as cancers in other patients, but research continues into the best way to manage cancers involved in LFS. Large prospective cohort studies are needed to select the best individualised treatment for every patient.

## Figures and Tables

**Table 1:** Results of published studies.

Study	Number of Patients	Receptor Status	Number of Patients Treated with Radiotherapy (Curative Setting)	Number of Ipsilateral Recurrences (Irradiated vs. Not)	Number of Contralateral Recurrences (Irradiated vs. Not)	Number of RIMs	RIM Histology	Median Follow-Up
Heymann et al., 2010 [29]	8	HR+: 6 HER2+: 2	6	3 vs. 0	4 vs. 1	2 (30%)	1 angiosarcoma 1 histiocytofibrosarcoma	6 years (2–13)
Petry et al., 2019 [30]	16	HR+: 8 HER2+: 6	12	0 vs. 2	0 vs. 0	2 (16.7%)	1 fibrosarcoma 1 leiomyosarcoma	4.375 years
Le et al., 2020 [31]	51	HR+: 23 HER2+: 22	18	1 vs. NA	0 vs. NA	1 (5.6%)	1 sarcoma (subtype not specified)	12.5 years (2–20)
Alyami et al., 2021 [32]	12 (21 primary breast tumors)	HR+: 8 HER2+: 10	5	2 (1 patient) vs. 1	2 (1 patient) vs. 2	0	None	7.3 years (0.67–18.5)
Petry et al., 2024 [33]	48	ER+: 38 PR+: 28 HER2+: 17	30	2 (6.7%) vs. 1 (5.6%)	3 (10%) vs. 2 (11.1%)	3 (10%)	NA	4.75 years
Sandoval et al., 2024 [34]	227	RH+: 140 HER2+: 87	79	NA	NA	6 (7.6% total, 4.8% risk at 5 years)	6 sarcomas (subtype not specified)	5.83 years

RIMs: radio-induced malignancies; NA: data not available; HR: hormone receptor positive; ER: estrogen receptor; PR: progesterone receptor; HER2: Human Epidermal Growth Factor Receptor-2.
